# Revealing the Character of Coordination Bonding in 2D Metal–Organic Frameworks

**DOI:** 10.1002/advs.202510414

**Published:** 2025-10-07

**Authors:** Dominik Brandstetter, Simone Mearini, Andreas Windischbacher, Yan Yan Grisan Qiu, Daniel Baranowski, Vitaliy Feyer, Claus Michael Schneider, Peter Puschnig

**Affiliations:** ^1^ Institute of Physics University of Graz Graz 8010 Austria; ^2^ Peter Grünberg Institute (PGI‐6) Jülich Research Centre 52428 Jülich Germany; ^3^ Faculty of Physics and Center for Nanointegration Duisburg‐Essen (CENIDE) University of Duisburg‐Essen 47048 Duisburg Germany; ^4^ Department of Physics and Astronomy UC Davis Davis CA 95616 USA; ^5^ Present address: Physical and Computational Sciences Directorate and Institute for Integrated Catalysis Pacific Northwest National Laboratory Richland WA 99354 USA

**Keywords:** metal–organic bond, metal–organic frameworks, photoelectron spectroscopy, photoemission orbital tomography

## Abstract

In this contribution, the coordination bond is investigated in a 2D metal–organic framework consisting of nickel and 7,7,8,8‐tetracyanoquinodimethane (TCNQ) on a Ag(100) substrate. Using angle‐resolved photoemission experiments supported by density functional theory calculations, the bonding is characterized as a result of the hybridisation between the ligand orbitals and the metal *d*‐states. Unambiguous experimental fingerprints are presented for such a mechanism by revealing the splitting in energy of the frontier molecular orbitals into sets of bonding/antibonding states in the 2D metal–organic framework. Furthermore, a qualitative analysis of the energy level alignment is given for the transition metal inside the coordination environment and discuss the role of the network structure on the formation of such hybrid states.

## Graphical abstract

Photoemission‐derived momentum maps and density functional theory calculations show the re‐appearance of frontier molecular orbital patterns at two distinct binding energies, a result of the formation of bonding and antibonding states below the Fermi edge from covalent hybridisation with the metal centres. The existence of hybrid states in the first Brillouin zone depends on the symmetry of the involved orbitals as well as the network structure.

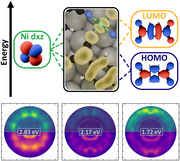

## 1 Introduction

In the past decade, 2D metal–organic frameworks (MOFs) have drawn significant attention as a new class of versatile materials.^[^ ^1^, ^2^, ^3^, ^4^, ^5^, ^6^ ^]^ Arguably one of their biggest benefits over other conventional 2D materials is that, by strategically choosing the constituents and carefully controlling the fabrication parameters, their electronic, magnetic and catalytic characteristics can be tuned.^[^ ^7^, ^8^, ^9^ ^]^ This is because these properties are dictated by the interactions between metal centres and organic ligands, which self‐assemble into periodic and ordered structures.^[^ ^10^, ^11^, ^12^, ^13^, ^14^, ^15^, ^16^, ^17^ ^]^ The design and optimisation of the metal‐organic network relies on a precise understanding of the nature of the electronic states formed by the hybridisation of metal *d*‐orbitals and ligand molecular orbitals. Conventionally, these hybridised states have been analysed through models such as ligand field theory, which describes the bonding interactions between metal centres and ligands based on their symmetries and the spatial overlap of the orbitals. Although a well established theory in the study of metallo‐organic complexes, clear experimental proof and the influence of the energy level alignment, or an orbital's tendency to attract electrons, on the character of the covalent bonding has so far not been established in the context of 2D coordination frameworks.

Here, we tackle this shortcoming by combining density functional calculations with angle‐resolved photoemission experiments in the joined framework of photoemission orbital tomography (POT). By making use of the energy‐resolved photoemission momentum maps, we are able to unambiguously reveal signatures of covalent bonding between the organic ligands and the transition metal *d*‐states, identifying the momentum space fingerprints of the chemical interaction as bonding and antibonding combinations of the involved orbitals. Our results also exhibit the characteristic energy splitting between the bonding and antibonding states not accounted for by mere molecular dispersion. We show that this splitting is driven not only by the symmetry of the interacting orbitals but also by the energy level alignment of the transition metal's *d*_*xz*_ orbital and the TCNQ ligand's former highest occupied molecular orbital (HOMO) and lowest unoccupied molecular orbital (LUMO). In the case of the LUMO, the relatively lower orbital energy of the Ni *d*_*xz*_ orbital stabilises the bonding state, while the TCNQ LUMO elevates the antibonding state. As such, this work provides clear and direct confirmation of textbook quantum mechanics in action.

## 2 Results and Discussion

We demonstrate our approach using the example of a 2D Ni‐TCNQ MOF, whose charge transfer characteristics with the underlying substrate have been the focus of a recent study.^[^ ^11^ ^]^ First, TCNQ molecules are deposited onto a Ag(100) surface, resulting in the formation of a highly ordered self‐assembled monolayer (SAM). Upon subsequent deposition of Ni atoms, the adsorbate layer undergoes a structural transition, leading to an extended 2D MOF. Note that, depending on the amount of deposited Ni, two stable MOF phases have been documented in the literature, which differ in the number of cyano groups coordinated to transition metal atoms.^[^ ^11^, ^18^, ^19^, ^20^ ^]^ For the purpose of this work, we will focus our discussion on the fully saturated Ni_1_(TCNQ)_1_ phase, with data for the Ni_1_(TCNQ)_2_ phase in Figures S1– S3 (Supporting Information). A more detailed comparison of their structural differences can be found in a recent publication by Mearini et al.^[^ ^11^ ^]^ Importantly, we want to stress that our conclusions are valid for both phases and can also be extended to other 2D MOFs.

**Figure 1:**
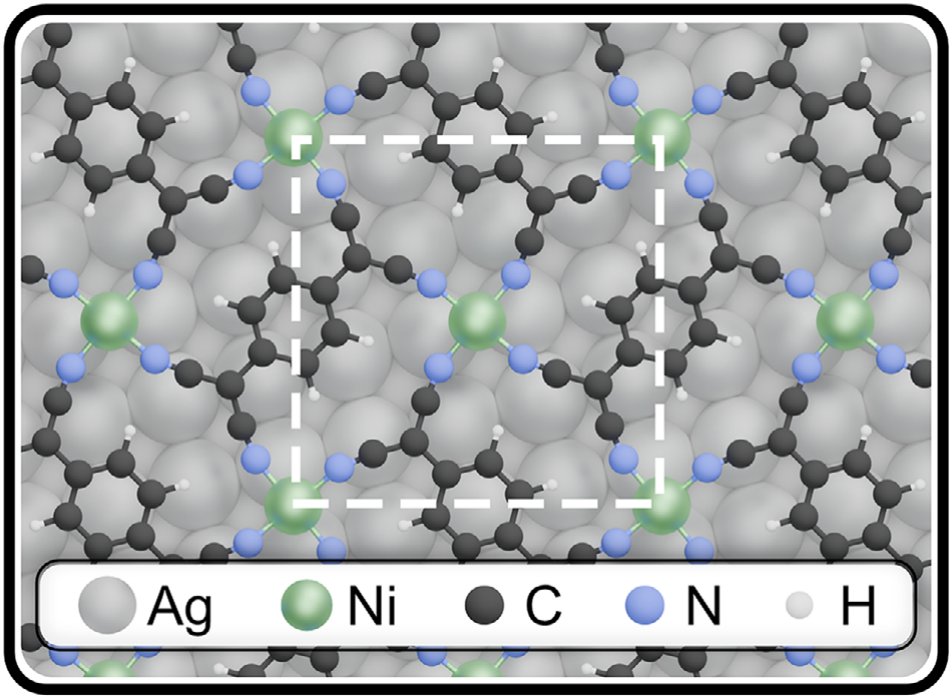
Structural model of a 2D monolayer of Ni_1_(TCNQ)_1_ on a Ag(100) substrate in the high coverage phase. Dashed white line indicates the unit cell.

**Figure** **1** shows a structural model of Ni_1_(TCNQ)_1_ on an Ag(100) surface, which is based on STM data from the literature^[^ ^18^ ^]^ as well as LEED experiments reported in a previous publication.^[^ ^11^ ^]^ In the same study,^[^ ^11^ ^]^ the oxidation state Ni(I) within the Ni‐TCNQ MOFs on Ag(100) has been experimentally clarified using NEXAFS. It has to be noted that, out of the two symmetry‐plausible adsorption sites, our tests have shown that Ni atoms favour the bridge site between two Ag atoms of the topmost substrate layer by 33meV per molecule over Ni atoms directly above Ag atoms. However, we found the effect of the adsorption site on the electronic structure to be negligible and will, therefore, restrict ourselves to the “bridge” adsorption geometry only, with data for the “top” site in Figure S4b (Supporting Information).

**Figure 2:**
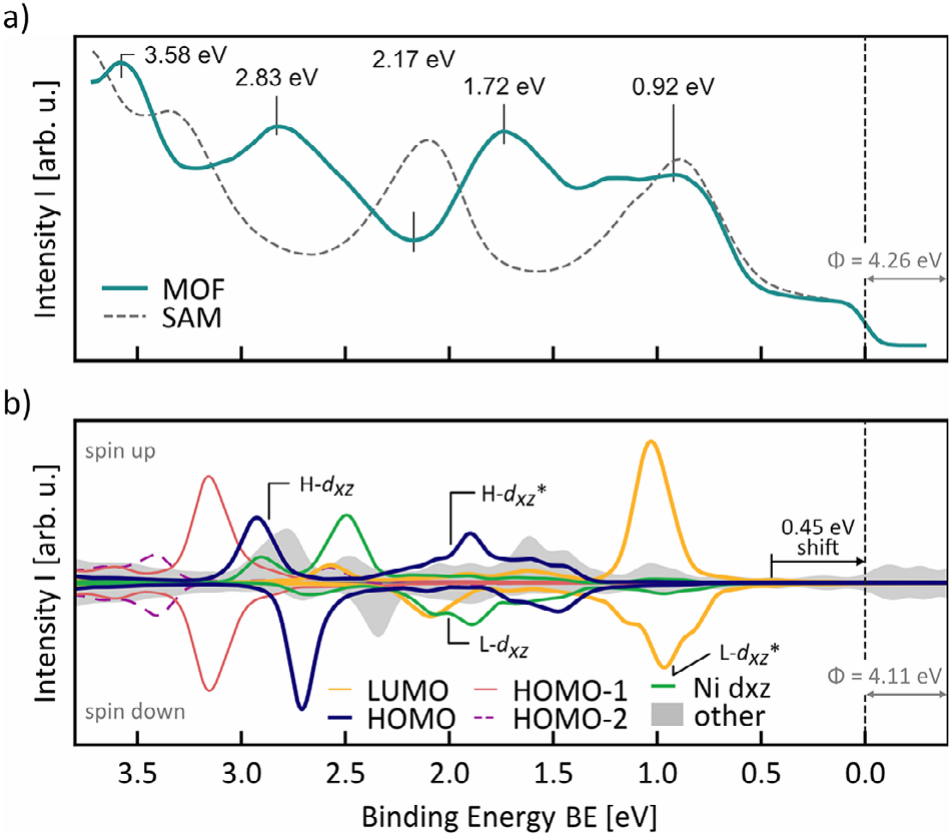
a) Experimental angle‐integrated valence band photoemission spectrum (photon energy 30 eV, p‐polarised light) for the SAM (grey dashed line) and the MOF (teal solid line) with prominent peaks labelled by their energy position. (b) Simulated density of states projected onto selected atomic and molecular orbitals (lines) rigidly shifted by 0.45eV toward higher binding energies (see main text). Projections onto the HOMO‐35 to HOMO‐3 and LUMO+1 to LUMO+11 are summed up as “other” (shaded area). The energy positions of the Bloch states for the bonding and antibonding combinations of the ligand HOMO and LUMO are indicated as H‐*d*_*xz*_ / H‐*d*_xz_^*^ and L‐*d*_*xz*_ / L‐*d*_xz_^*^, respectively.

In order to directly probe the electronic structure of the MOF, we use the momentum microscope at the NanoESCA beamline of the Elettra synchrotron light source in Trieste, Italy (details in Supporting Information), giving us access to the full photoemission intensity *I*(*BE*; *k*_*x*_, *k*_*y*_) for all binding energies *BE*. We first focus on the energy dependence of the photocurrent by integrating over all emission angles, that is, over the parallel momenta components *k*_*x*_ and *k*_*y*_, respectively. **Figure** **2**a shows the valence band spectrum *I*(*BE*) obtained for Ni_1_(TCNQ)_1_ (solid line) as well as the SAM (dashed line) as reference. Owing to the charge transfer from the Ni centres and the substrate to TCNQ, which has been the subject of previous studies,^[^ ^10^, ^11^, ^21^ ^]^ we can tentatively assign the first peak at 0.92eV to the former LUMO of the organic molecule, while lower peaks presumably correspond to former HOMO‐x orbitals.

**Figure 3:**
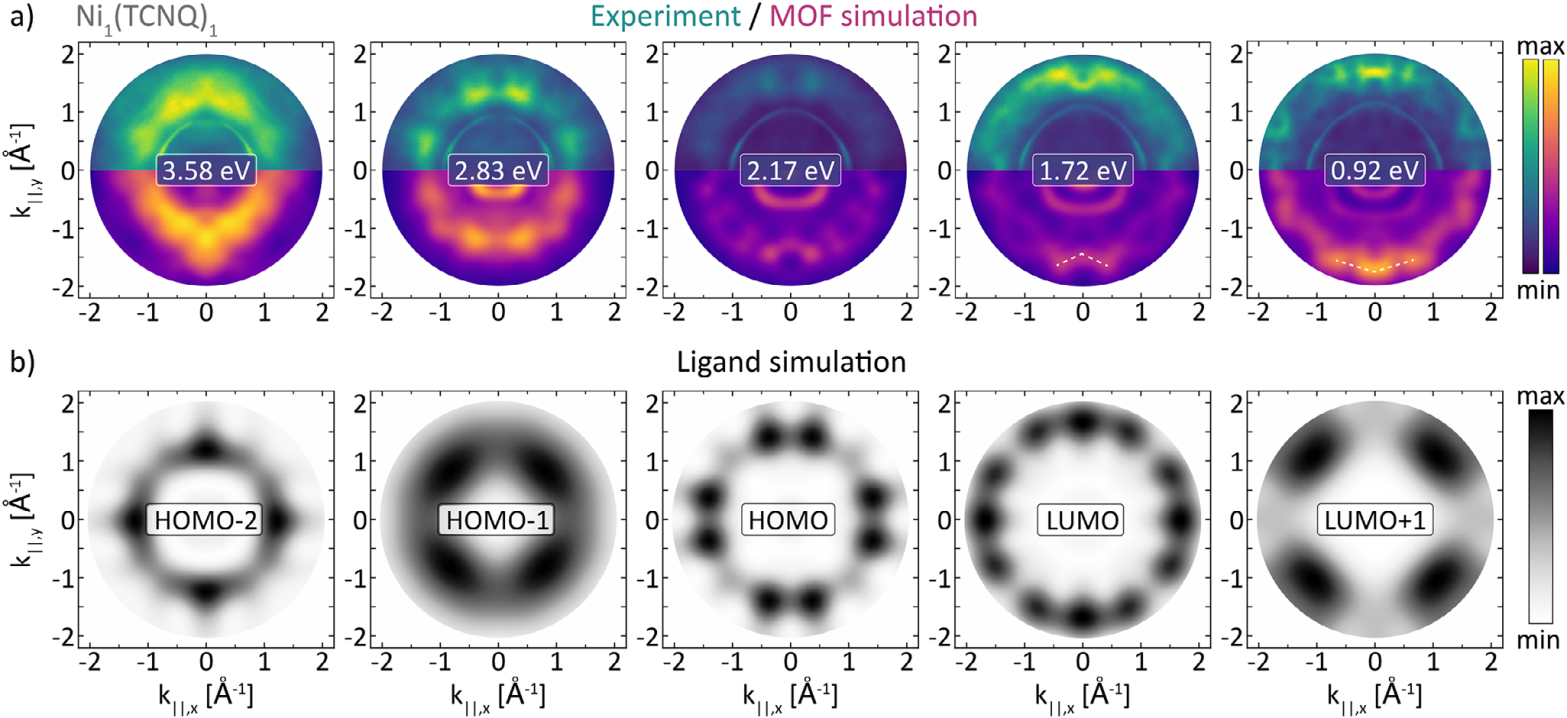
a) Experimental (top half, green) and simulated (lower half, purple) constant binding energy photoemission momentum maps of Ni_1_(TCNQ)_1_ on Ag(100) at the binding energy positions of the peaks in the valence band spectrum (Figure 2a). The momentum maps of the MOF simulation already account for a 0.45eV constant energy shift relative to the experimental data (see main text). A white dashed line in the simulated data indicates a triangle/inverse triangle shaped structure discussed in the main text. b) Momentum map patterns of the molecular frontier orbitals of a single free‐standing TCNQ molecule accounted for rotational and mirror domains of the substrate.

However, in order to unambiguously identify the underlying nature of these emissions, we utilise the unique momentum space fingerprints provided by the 3D data cube *I*(*BE*; *k*_*x*_, *k*_*y*_). Therefore, we extract constant energy momentum maps *I*(*k*_*x*_, *k*_*y*_) in **Figure** **3**a (top half) at the specific binding energies marked in Figure 2a. The bottom half of Figure 3a, then, compares the experimental data to a density functional theory based simulation of the photoemission intensity of the 2D periodic MOF layer including substrate in the photoemission orbital tomography formalism (“MOF simulation”). For details on the simulations, we refer to the Supporting Information.

Overall, we observe an excellent agreement between the MOF simulation and the experiment regarding all major molecular features located at *k*_||_ > 1.1 Å across all binding energies. Note that we rigidly shifted all calculated binding energies by ΔBE=0.45eV toward higher binding energies. Such a shift can be rationalised by the known limitations of interpreting Kohn–Sham energies from a GGA‐type calculation as electron extraction energies. Indeed, similar shifts of the GGA orbital energies compared to the experimental density of states have been documented also for other organic/metal interfaces.^[^ ^22^ ^]^ Except for this constant offset, both the energy ordering of all states and their relative energy positions are accurately predicted by the simulation. Finally, we note that, as already observed in previous investigations into other MOFs,^[^ ^10^, ^21^ ^]^ the ring‐shaped emission patterns originating from the *sp*‐bands of the Ag(100) substrate at *k*_||_ ⩽ 1.1 Å ^−1^ (experiment) / *k*_||_ ⩽ 0.6 Å ^−1^ (simulation) are not well captured in the simulations, which can be attributed to the limited number of substrate layers in the model. Nevertheless, a comparison between experimental and simulated data on the *BE* vs *k*_||_ dispersion for the clean substrate and the self‐assembled monolayer of TCNQ (Figure S6, Supporting Information) shows excellent agreement, further validating the simulation data.

A tentative assignment of the emission signatures of the extended system comes from the comparison to the simulated momentum maps for orbitals of the gas phase ligand (included in Figure 3b, “Ligand simulation”).^[^ ^23^, ^24^ ^]^ A common practice for such a comparison would be to fit a linear combination of gas phase orbitals to the experimental data cube *I*(*BE*; *k*_*x*_, *k*_*y*_), also known as orbital deconvolution.^[^ ^25^ ^]^ However, the presence of delocalised hybrid states in 2D MOFs induces a significant band dispersion,^[^ ^10^ ^]^ which results in an energy‐dependent modification of the momentum fingerprints of the orbitals, making a straight‐forward fit with static gas phase orbitals difficult. Despite these complication, the comparison with the gas phase momentum maps allows us to unambiguously identify the HOMO at BE=2.83eV and the LUMO at 0.92eV, and, furthermore, assess a mixture of HOMO‐2 and HOMO‐1 at 3.58eV.

While these mentioned assignments are straightforward, the momentum map at 1.72eV has more intricate features. Although it shows clear resemblance to the LUMO, both in comparison with the momentum map at 0.92eV as well as the Ligand simulation of the LUMO, there are some important deviations. The side peaks at 1.72eV are not only closer (*k*_*x*_ = ±0.4 Å ^−1^ compared to ±0.7 Å ^−1^) and stronger, they also appear at Δ*k*_*y*_ = 0.22 Å ^−1^ larger values than the central peak, matching the strong emission features of the HOMO. This leads to an upright triangle shape, unlike the flat or even inverse triangle shape seen in the LUMO map of the gas phase ligand (white dashed lines in the lower half of Figure 3a). While dispersive molecular emission features do shift in momentum space, in general, one can expect them to stay inside the patterns of the gas phase orbitals as they represent the spatially most localized (i.e., most delocalised in momentum space) possible electronic configuration. Furthermore, based on the MOF simulation, we can rule out any contributions from the LUMO+1 or other higher lying unoccupied orbitals.^[^ ^11^ ^]^ Therefore, we are left with the conclusion that the inverse triangle‐shaped feature must arise from a mixture between the central LUMO peak with the bright HOMO side peaks. Due to distinct dispersion relations for each orbital, they appear at different values of *k*_||_. This interpretation implies two important findings. First, the data suggests a broad band dispersion of the LUMO state in the valence region, which has been linked to the hybridisation of these frontier orbitals with the *d*‐states of the central transition metal atoms in other 2D MOFs.^[^ ^10^ ^]^ Second, the absence of any significant photoemission intensity at BE=2.17eV also implies a splitting of the HOMO contribution into two separate regions above and below 2.17eV.

Having established the accuracy of our MOF simulation, we can now rely on it to further investigate the contribution of individual molecular orbitals to the electronic structure imaged during the photoemission process. Figure 2b shows the spin‐resolved density of states projected onto molecular and atomic orbitals. Note that we have already included the aforementioned rigid shift of ΔBE=0.45eV. Figure 2b validates our previous identification of the molecular contributions and confirms the splitting of the HOMO projection (purple line) into two separate regions above and below ≈2.35eV. Furthermore, it also shows a splitting of the LUMO (orange line) with peaks for the spin down channel at 2.10eV and 0.97eV.

**Figure 4:**
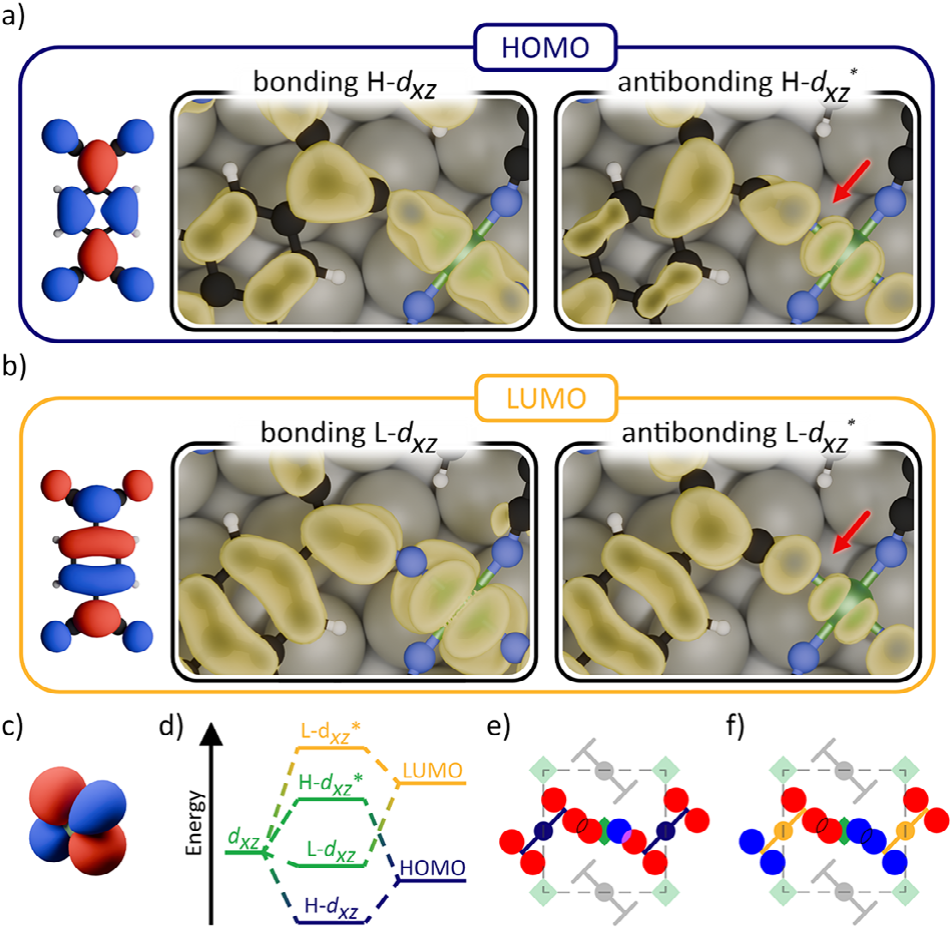
Real space representations of the gas‐phase molecular orbital (left) and the charge density (yellow) of the bonding (middle) and antibonding (right) hybrid Bloch functions with the Ni *d*_*xz*_ atomic orbital for a) former gas‐phase HOMO orbital at the *X*‐point and b) former gas‐phase LUMO orbital at the Γ‐point. The red arrow highlights the nodal plane for the antibonding states. c) Real space representation of the atomic Ni *d*_*xz*_ orbital. d) Qualitative schematic for the energy level alignment between a Ni *d*_*xz*_ orbital in a D_4h_ coordination environment and the molecular LUMO and HOMO. Schematic real space representation of a hypothetical Γ‐point Bloch state for the Ni *d*_*xz*_ and e) HOMO and f) LUMO hybrid state (green squares are Ni atoms; gray, cyan and purple tie‐fighter shaped structures represent TCNQ molecules; gray dashed line is the unit cell boundary; red and blue circles denote the sign of part of the real space wave function localised at the nitrile end groups of TCNQ and the Ni atom).

To understand the origin of this split, in **Figure** **4**a,b we take a look at the charge density (yellow) around the transition metal‐ligand coordination bond for four selected Bloch states labelled H‐*d*_*xz*_ / H‐*d*_xz_^*^ and L‐*d*_*xz*_ / L‐*d*_xz_^*^.^[^ ^26^ ^]^ These states were chosen because they maximise the product of the density of states projection onto the HOMO or former LUMO of a TCNQ and the Ni *d*_*xz*_. As such, they represent hybrid states with contributions from both, the molecular states and the Ni *d*‐state. For reasons that will be discussed later it is necessary to choose *X*‐point and Γ‐point Bloch functions for the HOMO and LUMO, respectively.

From these density plots, we can immediately recognise the bonding (H‐*d*_*xz*_, L‐*d*_*xz*_) and antibonding (H‐*d*_xz_^*^, L‐*d*_xz_^*^) combinations between the ligand molecular orbitals and the Ni *d*_*xz*_ state, which manifest in the presence (absence) of a nodal plane normal to the Ni–N bond (red arrow), at the intermediate region between the Ni and N atom. They can be easily understood in terms of a simple LCAO framework, as the two individual orbitals (HOMO/LUMO and *d*_*xz*_) give rise to two new orbitals (H/L‐*d*_*xz*_ and H/L‐*d*_xz_^*^) upon hybridisation. Hybridisation, in this context, is conceptualised as the prototypical covalent bond with the formation of a bonding/antibonding state pair out of two more primitive orbitals. Furthermore, their energy position marked in Figure 2b perfectly fits with the energy split in HOMO and LUMO projection discussed above, with the H‐*d*_*xz*_ at 2.87eV and L‐*d*_*xz*_ at 2.00eV aligned with the main projection peak at higher binding energies and the H‐*d*_xz_^*^ at 1.99eV and L‐*d*_xz_^*^ at 0.92eV at lower binding energies, respectively. The bonding states, i.e., in‐phase combination of the two orbitals, shift lower in energy (meaning higher binding energies) due to an excess amount of electron charge in the interstitial region. On the contrary, the antibonding states, i.e., the out‐of‐phase combinations, are raised in energy. The resulting energy splitting is also reflected in the photoemission data, explaining the double appearance of the molecular fingerprint in the photoemission maps discussed earlier.

Furthermore, in the present case of hetero‐nuclear hybridisation, the character of the bonding and antibonding states depends on the energy level alignment of the participating orbitals.^[^ ^27^ ^]^ Specifically, the electrophile bonding state will predominantly resemble the energetically lower original molecular or atomic orbital, whereas the higher in energy antibonding state will do the opposite. Based on this we can give a qualitative analysis of the energy level alignment relying on apparent trends of the absolute square of the projections onto the Ni *d*_*xz*_ and the molecular HOMO/LUMO of each of the four hybrid states listed in **Table** **1**.

**Table 1:** Absolute square of the projection of the hybrid states onto their primitive orbitals.

	Ni *d*_*xz*_	HOMO/LUMO
H‐*d*_*xz*_	0.134	0.208
H‐*d*_xz_^*^	0.167	0.206
L‐*d*_*xz*_	0.367	0.099
L‐*d*_xz_^*^	0.090	0.453

While the values from the projection onto atomic states^[^ ^28^, ^29^, ^30^, ^31^, ^32^, ^33^, ^34^ ^]^ and the projection onto molecular orbitals,^[^ ^35^ ^]^ listed in column two and three of Table 1 respectively, are not meaningful in isolation per se and cannot be compared directly, a clear trend is emerging. For the case of the HOMO based hybrid states, the projection onto the molecular states decreases going from bonding H‐*d*_*xz*_ to antibonding H‐*d*_xz_^*^ whereas the Ni *d*_*xz*_ projection increases. This is a clear indication that the bonding state is predominately based on the HOMO and the antibonding state has more of a Ni *d*_*xz*_ character, implying that the Ni *d*_*xz*_ inside this particular *D*_4*h*_ coordination environment is higher in energy than the molecular HOMO. At the same time, this trend is reversed and even stronger for the case of the LUMO based states, L‐*d*_*xz*_ and L‐*d*_xz_^*^, leading to the qualitative energy level alignment shown in Figure 4d. This represents an inversion of the orbital character usually expected in metal‐organic chemistry with a charge donation from the transition metal. The fact that both bonding and antibonding combination resemble molecular orbital features in the photoemission data (see Figure 3) can be rationalised by the small photoemission cross section of a single Ni atom compared to the molecular features and considering that the localised atomic *d*‐state orbitals give rise to a rather broad and featureless momentum space distribution that is difficult to detect experimentally.^[^ ^36^, ^37^ ^]^ We want to emphasise that, while a much more involved and sophisticated approach would be needed to make quantitative statements about the exact positions in energy of each state, our analysis can already provide a full picture of the energy level alignment. It clarifies how an individual transition metal *d*‐state, lifted out of degeneracy by a specific coordination environment, aligns relative to the energy position of molecular states. Importantly, this is not merely a simulated effect, but is based upon the good agreement to experimental data.

Lastly, we want to address the k‐point dependence mentioned earlier and highlight the role of the network structure on these bonding and antibonding states. Figure 4e,f shows schematics of the lattice periodic part of a hypothetical Bloch state for the case of a (e) HOMO‐*d*_*xz*_ and (f) LUMO‐*d*_*xz*_ hybrid states with the red and blue circles representing the positive and negative sign of the wavefunction lobes at the nitrile end groups and of the *d*_*xz*_ participating in the bond formation. Due to the particular structure of this MOF network, two nitrile end groups in trans position happen to be bonding with the same Ni atom (in the neighbouring unit cell) on opposite sides. Using a group theory approach based on symmetry adapted linear combination^[^ ^38^ ^]^ we can determine a *C*_2*h*_ point group with the representation of the Ni Γ(*d*_*xz*_) = *b*_*g*_. In the case of LUMO of TCNQ (Figure 4b,e) the two lobes in trans position differ in sign (one is red, the other is blue), which is perfectly in‐line with the lobes of the *d*_*xz*_ which also flip in sign (Γ(*L*) = *b*_*g*_). Thus, we can expect to see both the fully bonding and fully antibonding state at the Γ‐point where the Bloch factor *e* ^*ikx*^ = *e* ^*i*Γ*x*^ = 1 is constant across neighbouring unit cells. However, this is not the case for HOMO of TCNQ (Figure 4a,f) for which all four lobes at the nitrile end groups share the same sign (Γ(*H*) = *a*_*u*_) and thus no symmetry allowed combination exists. Figuratively speaking, at the Γ‐point the HOMO would need to be in a bonding and antibonding state with the same Ni at the same time. In order to find such a hybridisation we need to look at the boundary of the first Brillouin zone at the *X*‐point where the periodicity of the Bloch factor $e^{iXx}=e^{2i\pi \frac{x}{2}}$ spans two unit cells and therefore induces one additional sign flip in the entire Bloch function going from one unit cell to the next. We want to emphasise that this effect is a direct consequence of the particular network structure of the MOF in question and the symmetry of the orbitals involved. While important for the analysis of the character of the band, the overall bonding situation as well as all the photoemission data results from a summation over the entire Brillouin zone and is, thus, not directly apparent.

## 3 Conclusion

In summary, using the unique potential provided by the combined experimental and theoretical approach that is POT, we have been able to shed light on the nature of metal‐organic bonding in a 2D MOF. We unambiguously identified the splitting of the hybridised orbitals into bonding and antibonding combinations of the primitive orbitals of the constituents, a key characteristic of covalent bonds already predicted by the earliest theories of molecular orbital theory. We emphasise that, while we relied on the results of the DFT calculations for the definitive proof that this metal‐organic bond has a strong covalent‐like character, the experimental momentum space fingerprint of the HOMO splitting at 2.83eV and 1.72eV in Figure 3 is already a clear indication of this and represents the first time that bonding / antibonding states have been experimentally imaged using POT in the context of 2D MOF. We discussed how the symmetry of the frontier ligand orbitals together with the specifics of the network structure dictates the bonding formation inside the MOF. Lastly, based on the relative character of the bonding vs. antibonding states, we were able to resolve the relative energy ordering between a molecular orbital and a Ni *d*‐state inside a square planar ligand field and give a qualitative energy level alignment.

## Conflict of Interest

The authors declare no conflict of interest.

## Supporting information

Supporting Information

## Data Availability

The data that support the findings of this study are available from the corresponding author upon reasonable request.
