# Supplementary material for: Revealing the Character of Coordination Bonding in 2D Metal–Organic Frameworks
Source: Adv Sci (Weinh). 2025 Oct 7;12(47):e10414. doi: 10.1002/advs.202510414 (PMC12713051; doi:10.1002/advs.202510414)
Supplement: Supplementary file 1 — Supporting Information [file ADVS-12-e10414-s001.pdf]

Supplementary Information

Experimental details

Momentum microscopy experiments were performed at the NanoESCA beamline of the Elettra synchrotron light source in Trieste, Italy, using a photoelectron emission microscope (PEEM)[1] with a p-polarised photon beam ($\hbar\nu = 30\,\text{eV}$) at incident angle of 65°. With a pressure below 1×10^{-10} mbar at 90 K, an energy resolution of 100 meV and a momentum resolution of $\pm 0.05\,\text{Å}^{-1}$ are achieved. Varying the detection energy for the escaping photoelectrons allows for the collection of intensity I across a large binding energy range BE from the Fermi edge down to the onset of the Ag d-bands while simultaneously capturing also the full momentum dependence of the photoemitted electrons leading to a three-dimensional data-cube $I(BE, k_x, k_y)$.

MOF simulation details

Based on structural model (see Figure 1, main text), we perform density functional theory (DFT) calculations using VASP 5.4.4.,[2–8] where we employ a repeated slab approach with five layers of Ag and a 25 Å vacuum layer between periodic images of the slab. For the geometry optimisation, we allow all atoms to relax, except for the bottom three Ag layers, while using the PBE-GGA functional with van-der-Waals corrections according to the Grimme-D3 scheme with a Becke-Jones damping.[9,10] The self-interaction error in the strongly localised d-shell orbitals of the transition metal atoms is mediated by utilising a Hubbard U parameter in the Dudarev ansatz with $U = 3\,\text{eV}$.[11] The reciprocal lattice is sampled with a Γ-point centred 4x4x1 grid for the geometry optimisation and 10x10x3 grid for a final single-shot calculation for the electronic structure analysis. We simulate the photoemission momentum maps in the photoemission orbital tomography (POT) formalism including an exponential damping ($\gamma = 0.75\,\text{Å}^{-1}$) factor to mimic the mean free path of the escaping electrons.[12,13] The simulation data is publically available on the central NOMAD server hosted by FAIRmat accessible under the following DOI: https://dx.doi.org/10.17172/NOMAD/2025.02.25-3.

Data for the low coverage $Ni_1(TCNQ)_2$

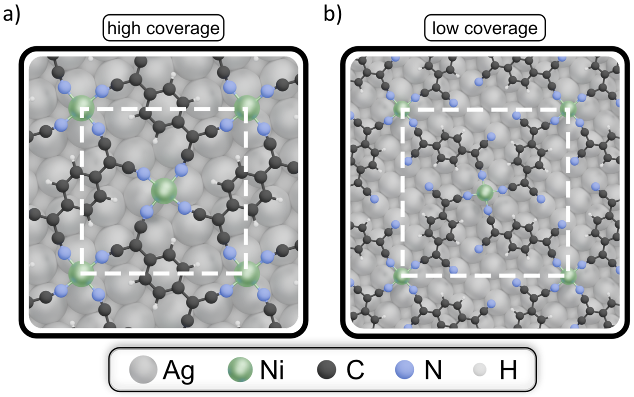

Figure S1. Comparison between the structural model of the 2D monolayer of (a) $Ni_1(TCNQ)_1$ (high coverage phase) and (b) $Ni_1(TCNQ)_2$ (low coverage phase) on a Ag(100) substrate. Dashed white line indicates the unit cell.

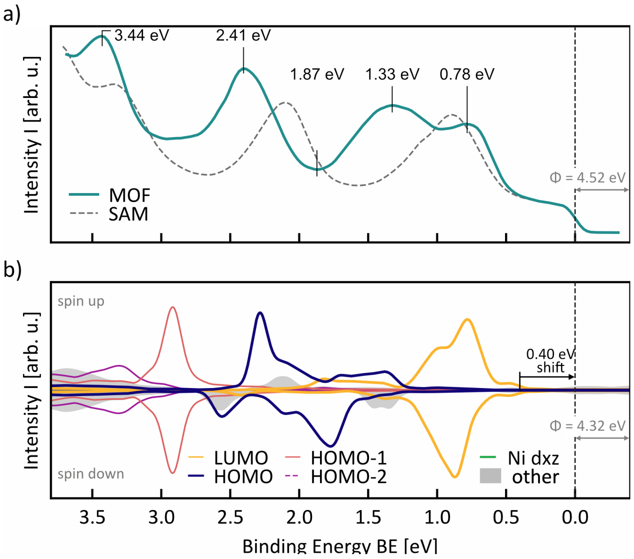

Figure S2. (a) Experimental angle-integrated valence band photoemission spectrum for the SAM (grey dashed line) and the $Ni_1(TCNQ)_2$ MOF (teal solid line) with prominent peaks labelled by their energy position. (b) Simulated density of states projected onto selected atomic and molecular orbitals (lines) rigidly shifted by 0.40 eV towards higher binding energies. Projections onto the HOMO-11 to HOMO-3 and LUMO+1 to LUMO+5 are summed up as "other" (shaded area).

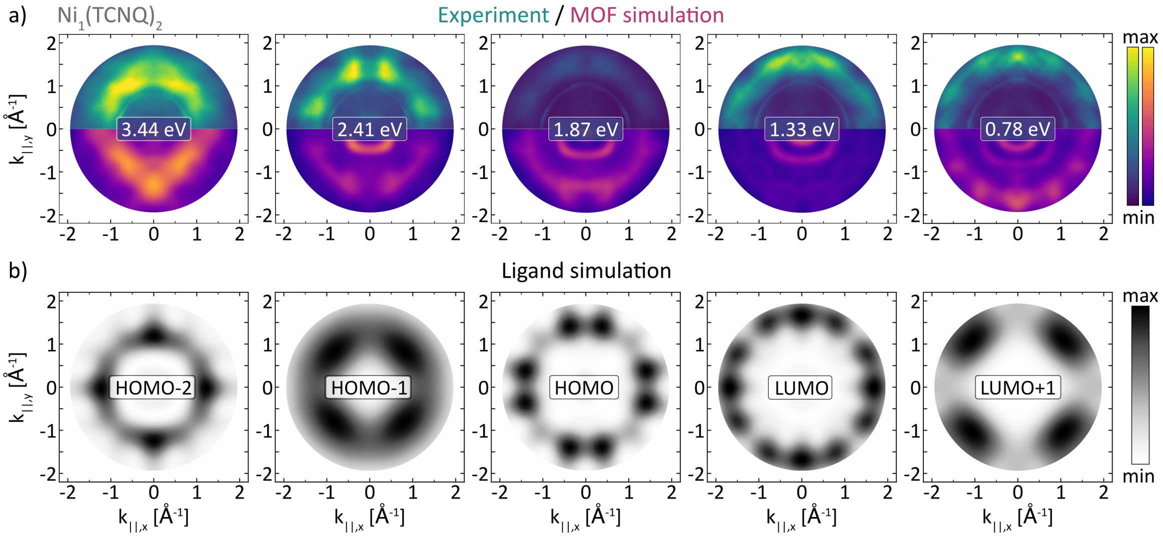

Figure S3. (a) Experimental (top half, green) and simulated (lower half, purple) constant binding energy photoemission momentum maps of $Ni_1(TCNQ)_2$ on Ag(100) at the binding energy positions of the peaks in the valence band spectrum (Figure 2a). The momentum maps of the MOF simulation already account for a $0.40\,\mathrm{eV}$ constant energy shift relative to the experimental data (see main text). (b) Momentum map patterns of the molecular frontier orbitals of a single free-standing TCNQ molecule accounted for rotational and mirror domains of the substrate.

Comparison between adsorption geometries for the high coverage $Ni_1(TCNQ)_1$ MOF

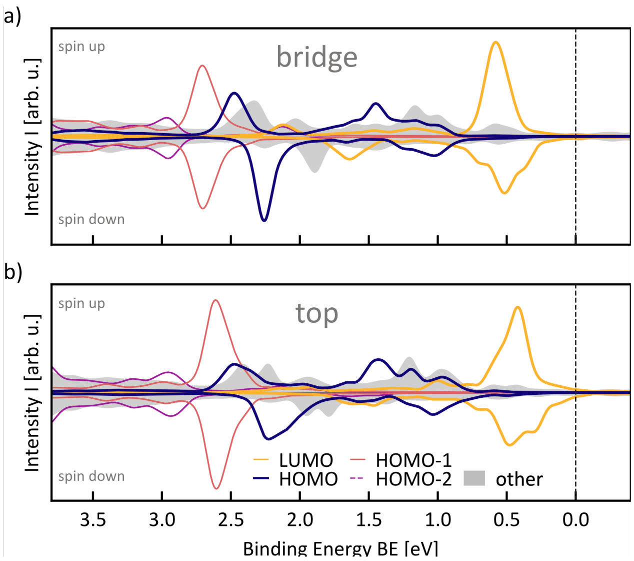

Figure S4. Simulated density of states projected onto selected atomic and molecular orbitals (lines) for (a) the energetically favoured bridge adsorption geometry and (b) the top geometry. Projections onto the HOMO-11 to HOMO-3 and LUMO+1 to LUMO+5 are summed up as "other" (shaded area). Notice that we did not include a rigid shift in energy for either geometry.

Atomic orbital projected density of states

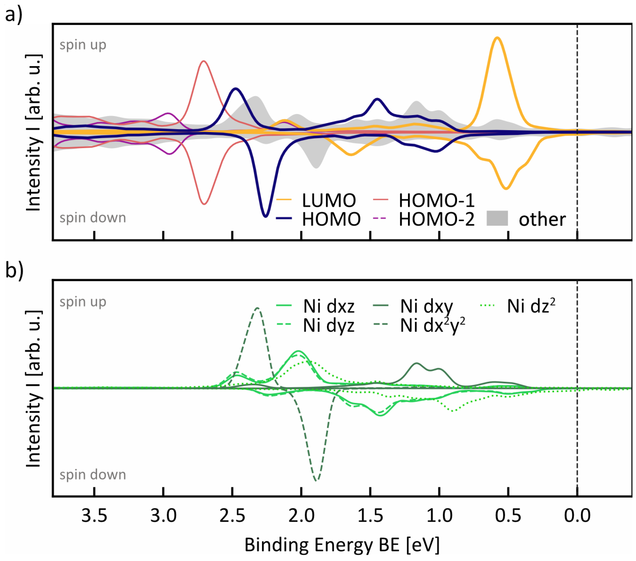

Figure S5. Simulated density of states of the low coverage $Ni_1(TCNQ)_1$ MOF in the bridge geometry projected onto (a) selected molecular orbitals and (b) the Ni d-states. Projections onto the HOMO-11 to HOMO-3 and LUMO+1 to LUMO+5 are summed up as "other" (shaded area). Notice that we did not include a rigid shift in energy projection.

Bandmap

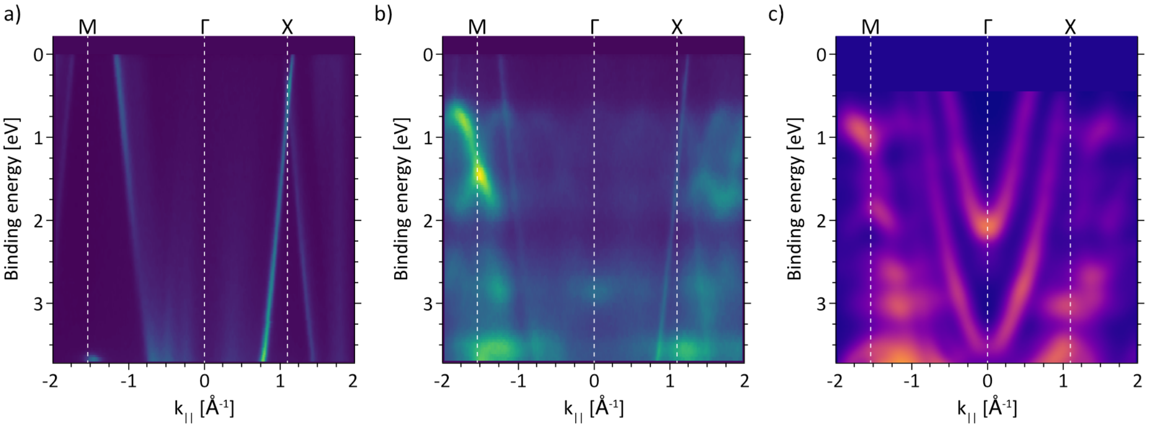

Figure S6. Bandmaps along the M-Γ-X direction of the first Brillouin zone of MOF layer. (a) Experimental bandmap for the clean Ag(100) surface. (b) Experimental bandmap for the MOF layer. (c) Simulated bandmap for the MOF layer shifted by $0.45\,\mathrm{eV}$ (see main text).

Photoemission data for the self-assembled molecular monolayer

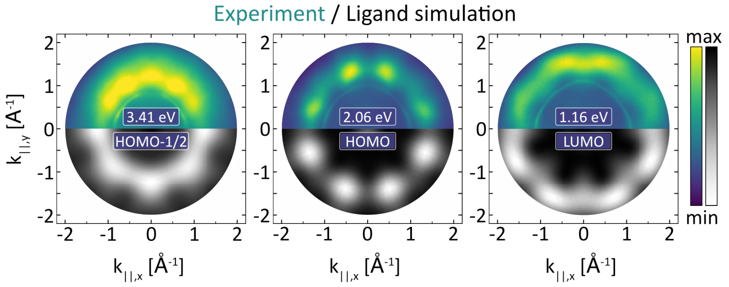

Figure S7. Photoemission momentum maps for the self-assembled molecular monolayer of TCNQ on Ag(100). (a) Experimental data at three selected binding energies corresponding to the first three peaks in the valence band spectrum. (b) Simulated momentum maps in the framework of photoemission orbital tomography (POT) of the frontier Kohn-Sham orbitals of TCNQ accounting for orientation and symmetry equalent growth domains of the substrate.[12,14,15]

References

[1] C. M. Schneider, C. Wiemann, M. Patt, V. Feyer, L. Plucinski, I. Krug, M. Escher, N. Weber, M. Merkel, O. Renault, N. Barrett, *Journal of Electron Spectroscopy and Related Phenomena* **2012**, *185*, 330.

[2] G. Kresse, J. Hafner, *Physical Review B* **1993**, *47*, 558.

[3] G. Kresse, J. Hafner, *Physical Review B* **1994**, *49*, 14251.

[4] G. Kresse, J. Furthmüller, *Computational Materials Science* **1996**, *6*, 15.

[5] G. Kresse, J. Furthmüller, *Physical Review B* **1996**, *54*, 11169.

[6] G. Kresse, D. Joubert, *Physical Review B* **1999**, *59*, 1758.

[7] J. P. Perdew, K. Burke, M. Ernzerhof, *Physical Review Letters* **1996**, *77*, 3865.

[8] P. E. Blöchl, *Physical Review B* **1994**, *50*, 17953.

[9] S. Grimme, J. Antony, S. Ehrlich, H. Krieg, *The Journal of Chemical Physics* **2010**, *132*, 154104.

[10] S. Grimme, S. Ehrlich, L. Goerigk, *Journal of Computational Chemistry* **2011**, *32*, 1456.

[11] S. L. Dudarev, G. A. Botton, S. Y. Savrasov, C. J. Humphreys, A. P. Sutton, *Physical Review B* **1998**, *57*, 1505.

[12] P. Puschnig, S. Berkebile, A. J. Fleming, G. Koller, K. Emtsev, T. Seyller, J. D. Riley, C. Ambrosch-Draxl, F. P. Netzer, M. G. Ramsey, *Science* **2009**, *326*, 702.

[13] D. Lüftner, S. Weiß, X. Yang, P. Hurdax, V. Feyer, A. Gottwald, G. Koller, S. Soubatch, P. Puschnig, M. G. Ramsey, F. S. Tautz, *Physical Review B* **2017**, *96*, 125402.

[14] D. Brandstetter, X. Yang, D. Lüftner, F. S. Tautz, P. Puschnig, *Computer Physics Communications* **2021**, *263*, 107905.

[15] T.-C. Tseng, N. Abdurakhmanova, S. Stepanow, K. Kern, *The Journal of Physical Chemistry C* **2011**, *115*, 10211.
